# Coronary Microvascular Dysfunction Is Associated With Myocardial Ischemia and Abnormal Coronary Perfusion During Exercise

**DOI:** 10.1161/CIRCULATIONAHA.119.041595

**Published:** 2019-11-11

**Authors:** Haseeb Rahman, Matthew Ryan, Matthew Lumley, Bhavik Modi, Hannah McConkey, Howard Ellis, Cian Scannell, Brian Clapp, Michael Marber, Andrew Webb, Amedeo Chiribiri, Divaka Perera

**Affiliations:** 1 From The British Heart Foundation Centre of Research Excellence, Schools of Cardiovascular Medicine & Sciences (H.R., M.R., M.L., B.M., H.M., H.E., B.C., M.M., A.W., D.P.), King’s College London, United Kingdom.; 2 Biomedical Engineering & Imaging Sciences (A.C., C.S.), King’s College London, United Kingdom.

**Keywords:** coronary artery disease, exercise, microvascular angina, perfusion imaging

## Abstract

**Background:**

Coronary microvascular dysfunction (MVD) is defined by impaired flow augmentation in response to a pharmacological vasodilator in the presence of nonobstructive coronary artery disease. It is unknown whether diminished coronary vasodilator response correlates with abnormal exercise physiology or inducible myocardial ischemia.

**Methods:**

Patients with angina and nonobstructive coronary artery disease had simultaneous coronary pressure and flow velocity measured using a dual sensor-tipped guidewire during rest, supine bicycle exercise, and adenosine-mediated hyperemia. Microvascular resistance (MR) was calculated as coronary pressure divided by flow velocity. Wave intensity analysis quantified the proportion of accelerating wave energy (perfusion efficiency). Global myocardial blood flow and subendocardial:subepicardial perfusion ratio were quantified using 3-Tesla cardiac magnetic resonance imaging during hyperemia and rest; inducible ischemia was defined as hyperemic subendocardial:subepicardial perfusion ratio <1.0. Patients were classified as having MVD if coronary flow reserve <2.5 and controls if coronary flow reserve ≥2.5, with researchers blinded to the classification.

**Results:**

Eighty-five patients were enrolled (78% female, 57±10 years), 45 (53%) were classified as having MVD. Of the MVD group, 82% had inducible ischemia compared with 22% of controls (*P*<0.001); global myocardial perfusion reserve was 2.01±0.41 and 2.68±0.49 (*P*<0.001). In controls, coronary perfusion efficiency improved from rest to exercise and was unchanged during hyperemia (59±11% vs 65±14% vs 57±18%; *P*=0.02 and *P*=0.14). In contrast, perfusion efficiency decreased during both forms of stress in MVD (61±12 vs 44±10 vs 42±11%; both *P*<0.001). Among patients with a coronary flow reserve <2.5, 62% had functional MVD, with normal minimal MR (hyperemic MR<2.5 mmHg/cm/s), and 38% had structural MVD with elevated hyperemic MR. Resting MR was lower in those with functional MVD (4.2±1.0 mmHg/cm/s) than in those with structural MVD (6.9±1.7 mmHg/cm/s) or controls (7.3±2.2 mmHg/cm/s; both *P*<0.001). During exercise, the structural group had a higher systolic blood pressure (188±25 mmHg) than did those with functional MVD (161±27 mmHg; *P*=0.004) and controls (156±30 mmHg; *P*<0.001). Functional and structural MVD had similar stress myocardial perfusion and exercise perfusion efficiency values.

**Conclusion:**

In patients with angina and nonobstructive coronary artery disease, diminished coronary flow reserve characterizes a cohort with inducible ischemia and a maladaptive physiological response to exercise. We have identified 2 endotypes of MVD with distinctive systemic vascular responses to exercise; whether endotypes have a different prognosis or require different treatments merits further investigation.

## Abstract 2

Supplemental Digital Content is available in the text.

**Clinical Perspective**

**What Is New?**

- In patients with angina without obstructive coronary artery disease, diminished coronary flow reserve in response to pharmacological vasodilatation identifies those with a maladaptive physiological response to exercise and global myocardial ischemia.
- There are 2 distinct endotypes of microvascular dysfunction: functional and structural, with differing degrees of systemic disease involvement and distinct mechanisms of ischemia.

**What Are the Clinical Implications?**

- Patients with angina and nonobstructive coronary artery disease can have exercise pathophysiology and global myocardial ischemia; the measurement of coronary flow reserve will help to characterize this population.
- Not all microvascular dysfunction is mechanistically identical; distinct endotypes may have differing prognosis and warrant individualized therapies.
- Targeting specific mechanistic abnormalities may improve the poorer prognosis observed in this population and would need to be studied in therapeutic trials.

**Editorial, see p 1817**

More than 40% of patients with angina have nonobstructive coronary artery disease (NOCAD); the physiological basis of their symptoms remains elusive, and most are offered no specific therapy beyond reassurance.^1–3^ Coronary microvascular dysfunction (MVD), defined by diminished coronary flow reserve (CFR) in response to a pharmacological vasodilator, affects a large proportion of these patients and portends an increased risk of major adverse cardiovascular events.^4–7^ Augmentation of coronary blood flow in response to increased myocardial oxygen demand is achieved by vasodilation of resistance vessels, mediated by both endothelium-dependent and endothelium-independent mechanisms.^8,9^ Adenosine causes endothelium-independent vasodilation of most vascular beds, including the coronary circulation, and is the most common test used to diagnose MVD. However, patients with MVD manifest symptoms during physiological exercise, a process distinct from pharmacological vasodilatation. Using the technique of wave intensity analysis, which provides directional, quantitative, and temporal information on the waves that govern coronary flow, pharmacological stress and exercise have been shown to act in fundamentally different ways.^10^ Furthermore, during exercise, intramural compression is not uniform across the left ventricle (LV) with the subendocardial layer subjected to the highest pressure, associated with the greatest impedance. In patients with angina and NOCAD, subendocardial hypoperfusion during vasodilator stress correlates with ischemic symptoms and is used to identify the presence of inducible ischemia in this cohort.^11^

We addressed the hypothesis that MVD (defined as CFR <2.5) is associated with demonstrable ischemia and abnormal coronary physiology during exercise. We also explored the pathophysiological mechanisms of attenuated flow reserve in MVD.

## Methods

The data that support the findings of this study are available from the corresponding author on reasonable request.

### Study Population

**Figure 1.**
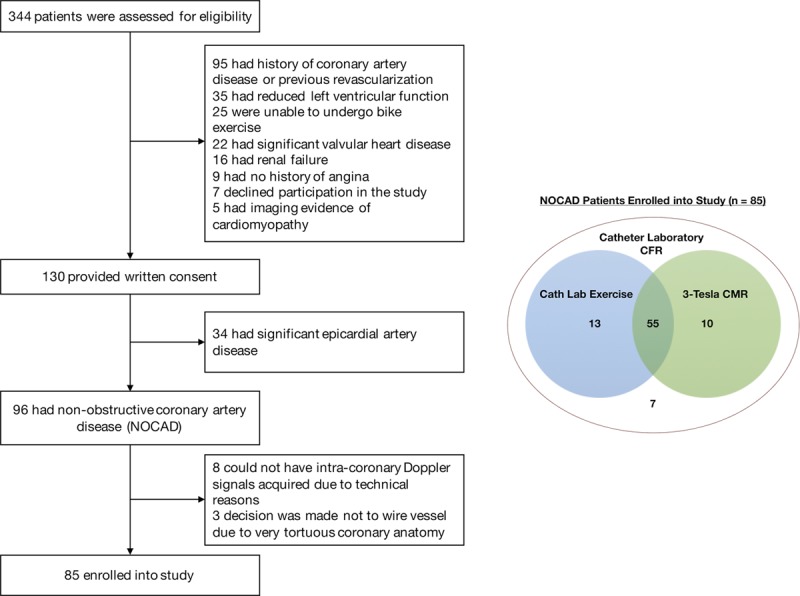
**Study screening criteria and tests.** Screening, and final study enrolment (left). Test completion for 85 patients enrolled into the study (right). Cath Lab Exercise indicates catheter laboratory supine bicycle exercise; CFR, coronary flow reserve; CMR, cardiac magnetic resonance imaging; and NOCAD, nonobstructive coronary artery disease.

Patients undergoing elective diagnostic angiography for investigation of exertional chest pain were enrolled in the study (Figure 1). Inclusion criteria were preserved LV systolic function (ejection fraction >50%) and unobstructed coronary arteries (<30% diameter stenosis and/or fractional flow reserve >0.80). Exclusion criteria were intolerance to adenosine, chronic kidney disease (estimated glomerular filtration rate <30 mL/min/m^2^), concomitant valve disease (greater than mild on echocardiography), recent acute coronary syndrome, cardiomyopathy, or any neuromuscular comorbidity that may affect their ability to perform bicycle exercise. Antianginal medications were stopped, and patients abstained from caffeine 24 hours before all study visits. Subjects gave written informed consent in accordance with the protocol approved by the UK National Research Ethics Service (17/LO/0203). The study was registered with the National Institute for Health Research UK Clinical Research Network portfolio database (Central Portfolio Management System identifier: 33170).

### Catheterization Protocol

**Figure 2.**
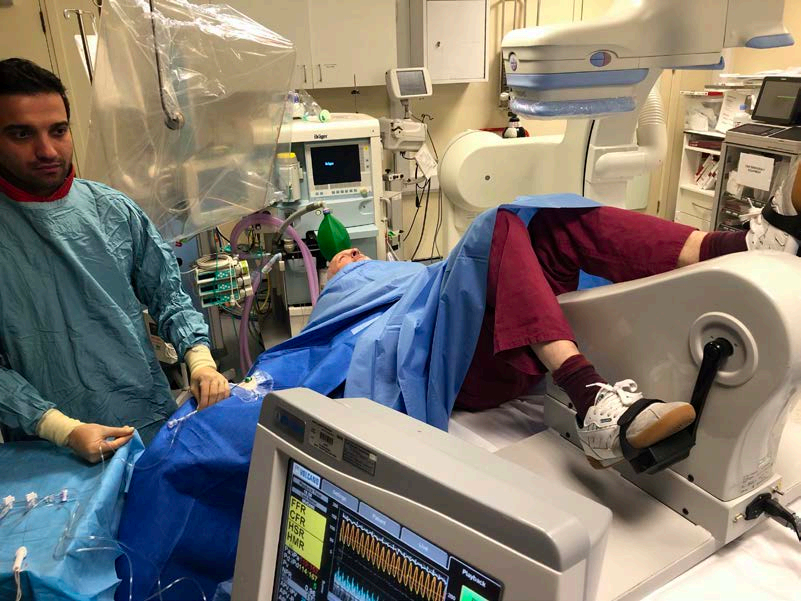
**Catheter laboratory set up during experimental exercise protocol.** The patient is cycling whilst catheterized via the right radial artery with the Combowire in the left anterior descending artery. The Combomap console (lower) is displaying continuous coronary pressure and flow velocity.

Catheterization was performed through the right radial artery using standard coronary catheters. All patients received 1 mg intravenous midazolam and intraarterial unfractionated heparin (70 U/kg) before intracoronary physiological measurements. A dual-pressure and Doppler sensor-tipped 0.014-inch intracoronary wire (Combowire, Philips Volcano, California) was used to measure distal coronary pressure and average peak flow velocity in the left anterior descending artery, as previously described.^10^ Hemodynamic measurements were recorded under resting conditions, during intravenous adenosine-mediated hyperemia (140 mcg/kg/min) and continuously during bicycle exercise, using a specially adapted supine ergometer (Ergosana, Bitz, Germany) attached to the catheter laboratory table (Figure 2). Exercise began at a workload of 30 W and increased every 2 min by 20 W. Where muscle weakness restricted increasing workloads, resistance was fixed at the maximum tolerated level and exercise continued until exhaustion. After full recovery from exercise or hyperemia, a second set of resting hemodynamic data was acquired before the final condition.

### Analysis of Coronary Physiological Data

Signals were sampled at 200 Hz, with data exported into a custom-made study manager program (Academic Medical Centre, University of Amsterdam, Netherlands) and analyzed on custom-made software, Cardiac Waves (Kings College London, UK). Microvascular resistance (MR) was calculated as distal coronary pressure divided by flow velocity for each condition. Hyperemic MR was dichotomously classified as normal (< 2.5 mmHg/cm/s) or elevated ≥2.5 mmHg/cm/s.^12^ Wave intensity was calculated as the product of the derivatives of distal coronary pressure and flow velocity, each with respect to time (d(distal coronary pressure)/dt × d(flow velocity)/dt), and wave separation performed as previously described.^13^ For each patient, 4 dominant waves were identified and included in our analysis: (1) backward compression wave (BCW), causing flow deceleration during isovolumetric contraction in early systole; (2) forward compression wave, causing flow acceleration, associated with peak aortic pressure; (3) forward expansion wave, causing flow deceleration associated with the fall in aortic pressure in late systole; and (4) backward expansion wave (BEW), causing flow acceleration during isovolumetric relaxation in early diastole. Perfusion efficiency was calculated as the percentage of accelerating wave intensity in relation total wave intensity, using areas under the respective curves.

Patients were classified offline as controls or more correctly as having normal CFR (CFR ≥2.5) and MVD (CFR <2.5) with researchers blinded to this classification throughout the study protocol.

### High-Resolution Cardiac Magnetic Resonance Imaging Protocol

All scans were performed on a dedicated 3-Tesla cardia magnetic resonance (CMR) scanner (Achieva, Philips Healthcare, Netherlands). Contiguous short-axis slices were acquired from the base to the apex to calculate LV function and mass (CVI42, v5.1.1, Circle Cardiovascular Imaging, Calgary, Ontario, Canada). After 3 minutes of intravenous adenosine (140 mcg/kg/min), stress perfusion data were acquired in 3 short-axis slices using a saturation-recovery *k-t* sensitivity encoding accelerated gradient-echo method.^14^ A dual-bolus gadobutrol (Gadovist, Bayer, Berlin, Germany) contrast agent scheme was used to correct for signal saturation of the arterial input function as previously described.^15^ Resting perfusion imaging was performed 15 minutes after stress, before acquisition of late gadolinium enhancement imaging (total contrast agent dose 0.2 mmol/kg).^16^ A proton density acquisition was performed before stress and rest acquisitions to correct for spatial inhomogeneities of surface coils.^17^ Before quantitative analysis, the perfusion images were motion corrected according to published methods.^18^ Quantitative analysis was performed as previously described by Fermi-constrained deconvolution.^19^ Myocardial blood flow estimates (MBF) were quantified in ml/min/g during rest and hyperemic stress; myocardial perfusion reserve was defined as the ratio between stress and rest perfusion. Endocardial-to-epicardial-perfusion ratios were calculated during hyperemic stress and during rest, by dividing stress MBF estimates within the inner and outer layers of myocardium. Inducible ischemia was defined as endocardial-to-epicardial perfusion ratio <1.0 during hyperemia (subendocardial relative hypoperfusion during pharmacological vasodilation).^11^

NT-proBNP (N-terminal pro-B-type natriuretic peptide) levels were measured before angiography using conventional clinical assays. Diastolic function was assessed using standard transthoracic echocardiography.

### Statistical Analyses

Sample size was estimated on the basis of the coprimary end points, exercise perfusion efficiency and frequency of ischemia. Assuming an allocation ratio of 1:1, 66 patients would be needed to detect a minimum difference in exercise perfusion efficiency of 12% (predicted SD, 17%) and 58 patients to detect a 50% relative decrease in the frequency of ischemia (predicted rate in MVD, 70%) at 80% power and 5% significance. To allow for potentially unequal allocation, data censoring as a result of quality issues, and incomplete datasets, we aimed to enroll 85 patients. Statistical analysis was performed using SPSS version 24 (IBM Corp., Armonk, New York). Normality of data was visually assessed (using histograms and the normal Q-Q plot) and using the Shapiro–Wilk test. Continuous normal data are expressed as mean±SD and compared using paired or unpaired Student *t* tests as appropriate. Nonnormal data are expressed as median±interquartile range and compared using Mann–Whitney *U* test, and categorical variables were compared with chi-square tests. Two-sided *P*-values <0.05 were considered nominally significant, with no correction for multiplicity of testing. Baseline variables found to correlate with exercise perfusion efficiency or inducible ischemia on univariate analysis (*P*<0.05) were assessed by a multiple linear regression model.

## Results

### Patient Characteristics

Eighty-five patients were recruited into the study (45 were classified as having MVD and 40 had normal CFR). Groups were well-matched for cardiovascular risk factors and preprocedural medications, whereas there were more women in the MVD group (87% vs 68%; *P*=0.03) (Table 1). All patients had resting and hyperemic coronary physiology measurements, 68 patients performed the full bicycle exercise protocol, and 65 underwent the 3-Tesla perfusion CMR protocol (Figure 1). The MVD and normal CFR groups exercised for the same duration (394±109 vs 408±146 s; *P*=0.67) and workload (63±27 vs 71±27 W; *P*=0.25).

**Table 1.**
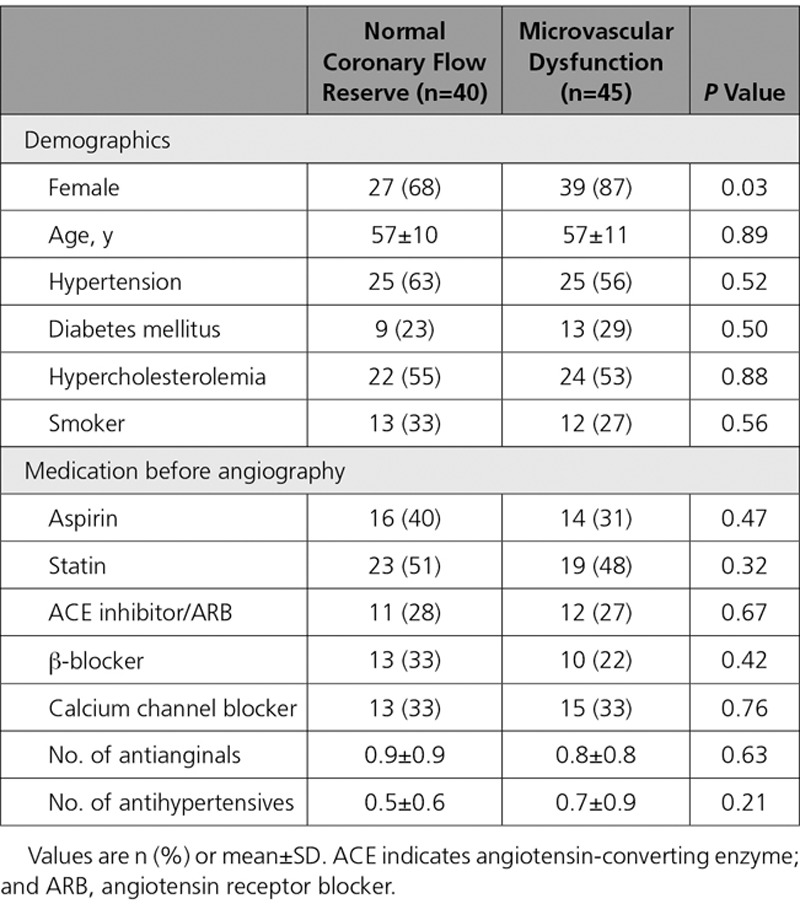
**Patient Characteristics and Long-Term Medication Before Angiography**

### Coronary Hemodynamic Data

CFR was 1.9±0.3 in the MVD group and 3.2±0.6 in the normal CFR group, with minimal epicardial disease in both groups (fractional flow reserve 0.92±0.05 vs 0.93±0.05, *P*=0.24). At rest, patients with MVD had reduced MR compared with those with normal CFR (5.3±1.9 vs 7.3±2.2 mmHg/cm/s; *P*<0.001) and higher resting coronary blood flow velocity (22.3±6.9 vs 15.0±4.7 cm/s; *P*<0.001) despite similar rate-pressure product between both groups (11 781±2929 vs 10 720±2634 bpm.mmHg; *P*=0.12). However, MR was similar in both groups during each form of stress (during exercise, 4.5±1.7 vs 4.7±1.6 mmHg/cm/s; *P*=0.49; during hyperemia, 2.4±0.8 vs 2.1±0.5 mmHg/cm/s; *P*=0.10).

**Figure 3.**
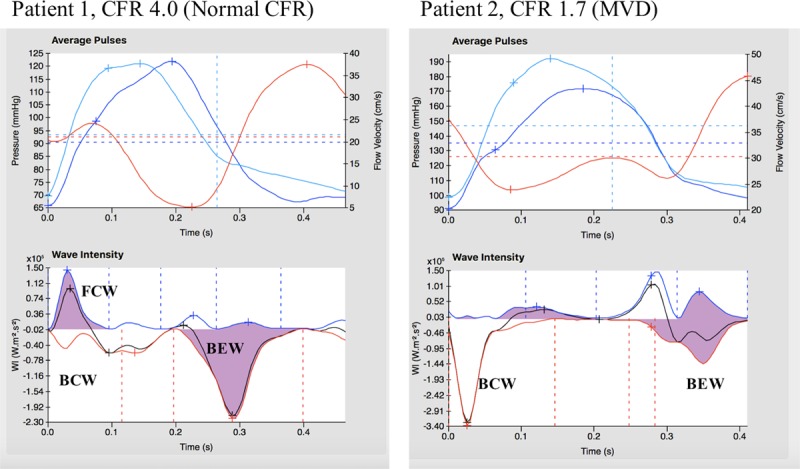
**Two examples of wave intensity analysis during peak exercise in a control patient with normal coronary flow reserve (CFR) (left) and microvascular dysfunction (MVD) patient (right).** Ensemble averaged aortic pressure (top, light blue), coronary pressure (top, dark blue), flow velocity (top red) and wave intensity analysis (bottom). BCW indicates backward compression wave; BEW, backward expansion wave; and FCW, forward compression wave.

**Figure 4.**
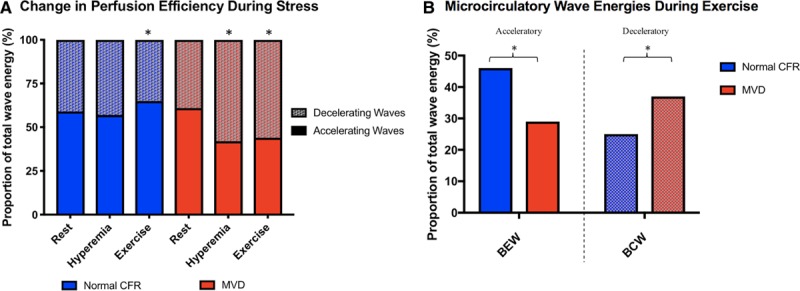
**Coronary wave intensity analysis during stress. (A)** During exercise, microvascular dysfunction (MVD) patients have a smaller proportion backward expansion wave (BEW) and greater proportion of backward compression wave (BCW) energy than patients with normal coronary flow reserve (CFR). *P<0.001. **(B)** In patients with MVD, the total percentage of accelerating wave intensity decreases in response to exercise and during vasodilator mediated hyperemia (reduced coronary perfusion efficiency) from rest; in those with normal CFR, coronary perfusion efficiency remains unchanged during exercise or hyperemia from resting conditions (*P<0.05).

By wave intensity analysis, both groups had similar profiles at rest. During exercise and hyperemia, the magnitude of all waves increased from rest, although the relative changes in accelerating and decelerating waves was significantly different in the 2 groups. Typical coronary pressure and flow waveforms, with corresponding wave intensity analysis profiles during peak exercise, are shown in Figure 3. In those with normal CFR, perfusion efficiency was enhanced during exercise (from 59±11% to 65±14%; *P*=0.02) and remained unchanged during hyperemia (57±18%; *P*=0.14) (Figure 4a). In contrast, in patients with MVD, perfusion efficiency decreased from rest (61±12%), during peak exercise (44±10%) and hyperemia (42±11%; *P*<0.001 for both conditions). The difference between normal CFR and MVD groups was mainly in the microcirculation-derived backward waves rather than in the aorta-derived forward waves; at peak exercise, patients with MVD had proportionately larger decelerating BCW (36±11 vs 24±12%; *P*<0.001) and smaller accelerating BEW (30±6 vs 46±12%; *P*<0.001) than did those with normal CFR (Figure 4b).

### Perfusion CMR

Sixty-eight patients attended for 3-Tesla CMR and 65 (38 with MVD, 27 with normal CFR) underwent the full protocol (3 were claustrophobic, preventing exam completion). MVD and normal CFR groups had similar LV ejection fraction (66±5% vs 64±6%; *P*=0.33) and indexed mass (41±11 vs 42±19 g/m^2^; *P*=0.77). There was no fibrosis or scar identified during late gadolinium enhancement in any patient. Patients with MVD had a lower myocardial perfusion reserve than did those with normal CFR (2.01±0.42 vs 2.66±0.42; *P*<0.001) and higher resting MBF (1.37±0.37 vs 1.13±0.20 ml/min/g; *P*=0.004). Stress MBF was similar between patients with MVD and patients with normal CFR (2.68±0.71 vs 2.98±0.54 ml/min/g; *P*=0.08). Patients with MVD had a lower stress endocardial-to-epicardial-perfusion ratio than did those with normal CFR (0.93±0.08 vs 1.05±0.11; *P*<0.001). As dichotomously predefined, the incidence of inducible ischemia was significantly higher in patients with MVD than in patients with normal CFR (82% vs 22%; *P*<0.001; Table 2).

**Table 2.**
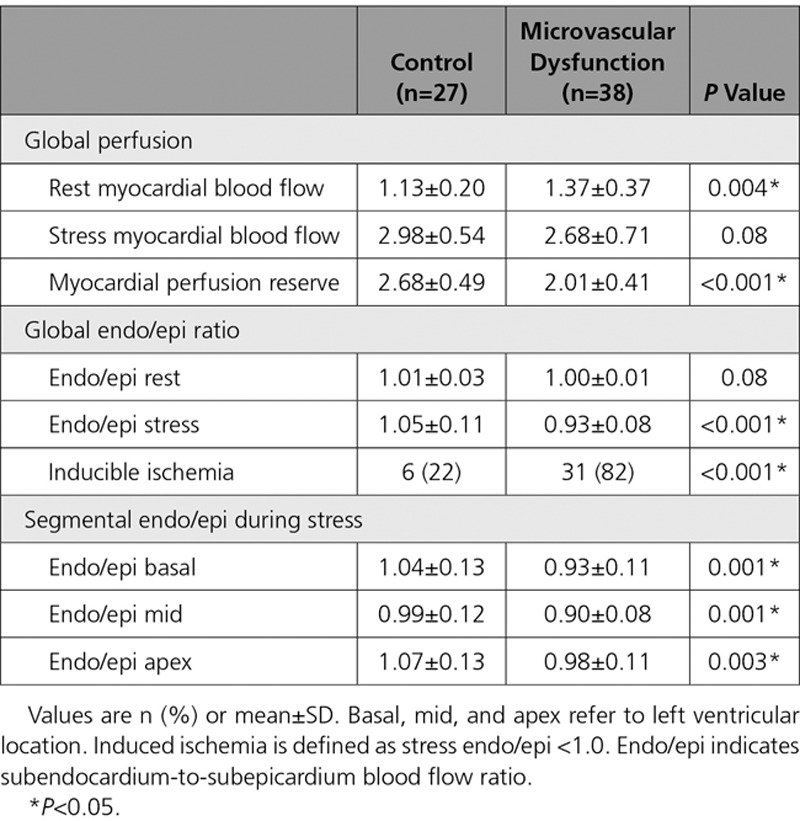
**Results From Quantitative Perfusion Cardiac Magnetic Resonance Imaging**

### Pathophysiological MVD Endotypes

**Figure 5.**
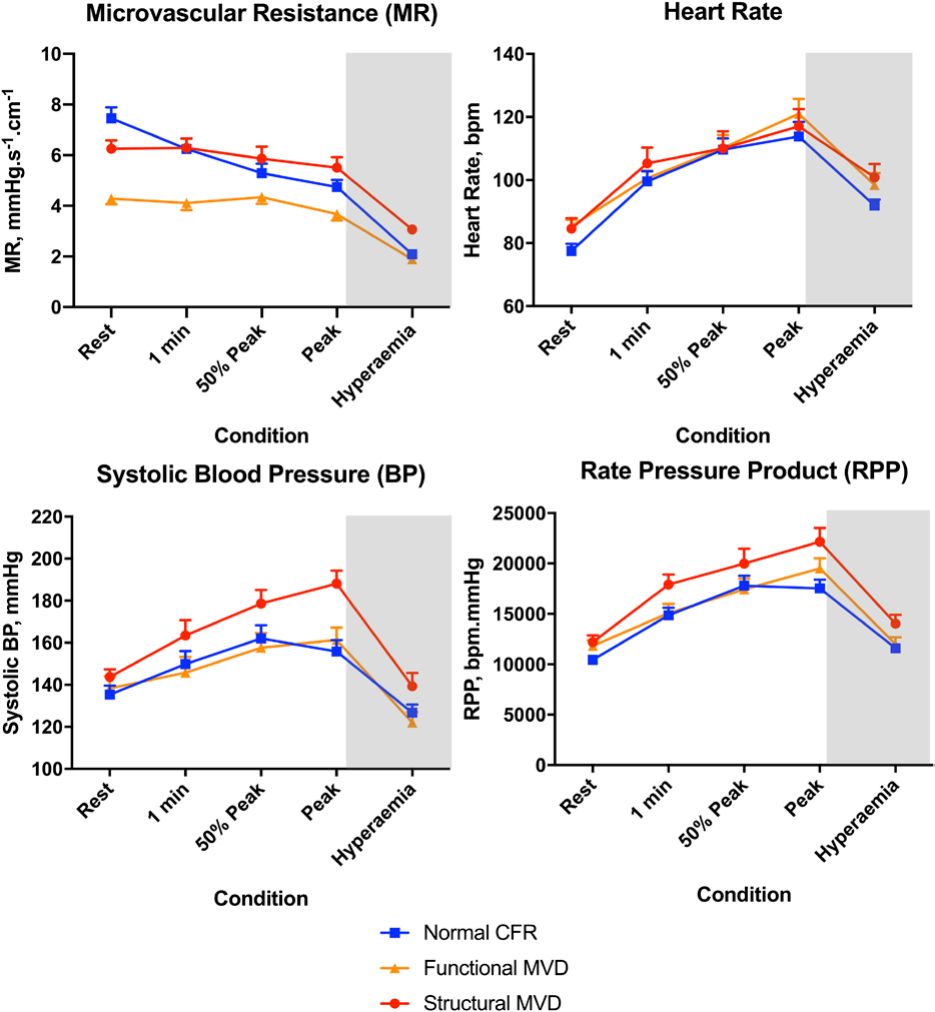
**Coronary and systemic hemodynamic responses to stress.** Time points are 1 minute after onset of exercise, 50% of maximal exercise time and peak (immediately before termination of exercise). White area denotes physical exercise, gray area denotes pharmacological hyperemia. bpm indicates beats per minute; CFR, coronary flow reserve; hyperemia, adenosine-induced hyperemia; and MVD, microvascular dysfunction.

Among the MVD cohort, 62% had functional MVD with normal minimal (hyperemic) MR, and 38% had structural MVD with elevated hyperemic MR. Patients with structural MVD had a higher incidence of hypertension and diabetes mellitus compared with patients with functional MVD (76% vs 43%; *P*=0.03; and 47% vs 18%; *P*=0.04). In patients with functional MVD, resting MR was reduced compared with patients with structural MVD and patients with normal CFR (4.2±1.0 vs 6.9±1.7 vs 7.3±2.2 mmHg/cm/s; *P*<0.001 for both). In patients with structural MVD, hyperemic MR was higher than in patients with normal CFR and patients with functional MVD (3.1±0.7 vs 2.1±0.5 vs 1.9±0.4 mmHg/cm/s; *P*<0.001 for both; Figure 5). Patients with normal CFR had a larger reduction in MR during exercise and hyperemia (2.9±2.0 and 5.3±2.1 mmHg/cm/s) than did structural MVD (0.8±1.0 and 3.2±1.1 mmHg/cm/s; *P*<0.001 for both) and functional MVD (0.8±1.0 and 2.4±1.0 mmHg/cm/s; *P*<0.001 for both) endotypes. In terms of external work, during peak exercise, the structural MVD group had a higher systolic blood pressure (188±2 5mmHg) than did the functional MVD (161±27 mmHg; *P*=0.004) and the normal CFR group (156±30 mmHg; *P*<0.001) and a higher rate-pressure product (22 157±5497 vs 19 519±4653 vs 17 530±4678 bpm.mmHg; *P*=0.12 vs functional and *P*=0.004 vs normal CFR; Figure 5). Perfusion efficiency during exercise and hyperemia were similar in patients with functional and structural MVD (46±9% vs 41±10% and 41±12% vs 44±9%; *P*=0.12 and *P*=0.31, respectively). Functional and structural MVD had similar myocardial perfusion reserve and stress endocardial-to-epicardial-perfusion ratio (2.01±0.41 vs 2.05±0.44; *P*=0.78; and 0.94±0.10 vs 0.93±0.05; *P*=0.66). The rate of inducible ischemia was numerically lower in functional MVD versus structural MVD, but this difference was not statistically significant (77% vs 88%; *P*=0.42).

NT-proBNP value was highest in patients with structural MVD compared with patients with functional MVD and patients with a normal CFR (132 [82–179] vs 69 [32–116] vs 34 [22–90] pg/ml; *P*=0.01 and *P*<0.001). Diastolic function measured by echocardiography tissue Doppler e/e’ was similar between structural MVD, functional MVD and patients with normal CFR (8.4±2.3 vs 7.5±3.0 vs 6.9±2.2; *P*=0.39 and *P*=0.07).

**Figure 6.**
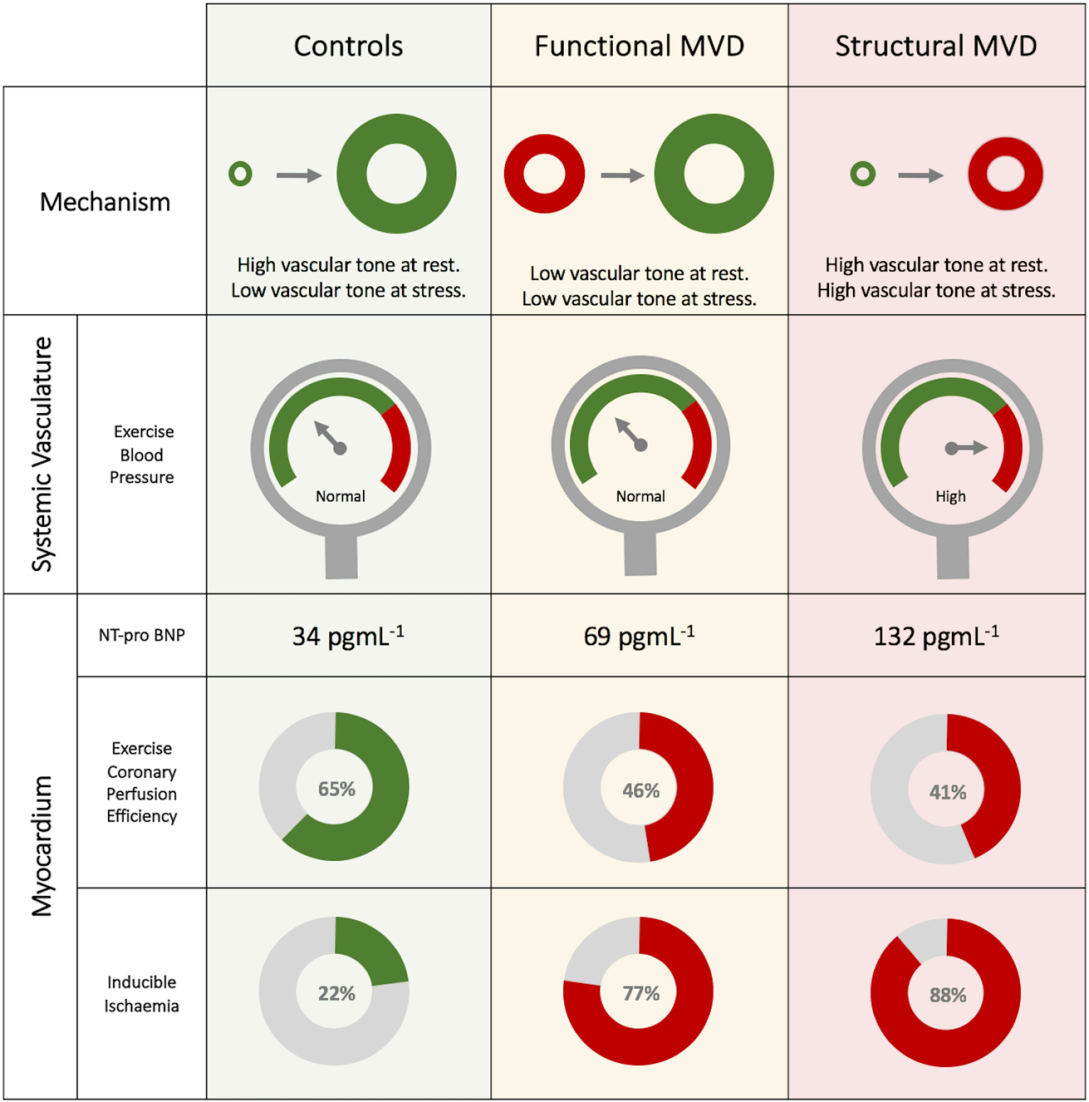
**Coronary flow reserve (CFR) measurement in patients with angina and nonobstructive coronary artery disease (NOCAD).** Low CFR can arise via 2 distinct microvascular dysfunction endotypes, classified using hyperemic microvascular resistance. Functional and structural microvascular dysfunction (MVD) endotypes have differing degrees of systemic disease involvement, however both display reduced coronary perfusion efficiency during stress and higher rates of global myocardial ischemia compared with controls with normal CFR.

The pathophysiological changes of diminished coronary flow reserve in NOCAD are summarized in Figure 6.

## Discussion

This is the first study to directly assess coronary blood flow during exercise in patients with microvascular dysfunction and compare these changes with high-resolution perfusion imaging. Among patients with angina and unobstructed coronary arteries, those with diminished coronary flow reserve have a higher prevalence of inducible myocardial ischemia and reduced global perfusion reserve in addition to reduced coronary perfusion efficiency during physical exercise compared with controls with preserved coronary flow reserve. We distinguished 2 microvascular dysfunction endotypes, those with low resting microvascular resistance (functional) and those with high minimal microvascular resistance (structural), both displaying similar myocardial perfusion characteristics and distinct exercise pathophysiology. The relationship with diminished coronary vasodilator reserve and these pathophysiological changes may underlie the poorer prognosis exhibited by patients with microvascular dysfunction and whether each endotype warrants individualized therapies will need to be studied further.

### Diminished Flow Reserve

The historical perception of MVD is that high vascular resistance during periods of increased demand or pharmacological vasodilatation is the key mechanistic abnormality. This structural MVD endotype may represent architectural changes such as capillary rarefaction and downstream myocardial hypertrophy and fibrosis. Indeed, there are several physiological parallels with heart failure with preserved ejection fraction, where coronary blood flow is elevated during rest and similarly diminished during vasodilator hyperemia.^20^ However, this state of raised minimal MR may be surmountable over time with appropriate treatment and so the term structural should not be considered to imply an irreversible disease process. Many authors have suggested that high minimal microvascular resistance is a more reliable tool for identification of patients with MVD and that this should replace measurements such as CFR, where resting conditions are integrated particularly in a population where there is mixed epicardial artery disease.^21^ However, in our NOCAD population, 62% of patients with MVD had preserved minimal MR (or functional MVD) despite a diminished CFR, yet they displayed a similar pathological phenotype as the traditional structural MVD group. The finding of low resting MR suggests that this is a functional disorder rather than being primarily the result of structural vascular changes leading to elevated minimal MR. This is consistent with recent observations by other groups where elevated myocardial and coronary blood flow was the likely mechanism of diminished flow reserve in MVD.^22,23^ In this context, elevated resting flow could be a response to increased myocardial oxygen demand or represent disordered autoregulation. We did not find a statistically significant difference in mechanical cardiac work at rest, as estimated by rate-pressure product, but it should be noted that myocardial oxygen demand is also dependent on basal metabolic activity and calcium cycling.^24^ High baseline MBF has been proposed to be due to increased myocardial oxygen consumption secondary to myocardial metabolic derangements, in studies of patients fitting the historical Cardiac Syndrome X definition.^25–27^ Diminished flow reserve in patients with diabetes mellitus has been shown to be due to elevated resting flow in the early stages of the disease.^22^ This may be a response to increased basal oxygen consumption as the metabolic shift in diabetics, from glucose to fatty acid oxidation, results in fewer ATP molecules produced per molecule of oxygen consumed; this theory would need to be explored further in MVD. While increased resting flow can lead to a reduction in vasodilator capacity, it may also play a role in disease progression. Chronically raised coronary blood flow may precipitate structural vascular changes, a process commonly found in other organs, such as the renal or pulmonary vascular beds.^28,29^ This may also explain the bimodal distribution of microvascular resistance found in patients with diabetes mellitus, with an early functional disorder progressing to structural changes.^22^ Whether functional MVD represents a precursor to structural MVD and early intervention may prevent disease progression is currently unknown. At present, no disease-modifying therapies exist specifically for MVD.

### The Mechanism of Ischemia

Within the structural MVD group, exercise-induced hypertension leads to increased myocardial oxygen demand during exercise. Attenuated reduction in afterload with exercise would interrupt the usual synergistic response of the coronary and peripheral circulations and predispose to ischemia; in theory, large vessel vasodilators may enhance the normal synergistic adaptation to exercise preferentially among this disease endotype.^30^ The structural MVD group have more established cardiovascular risk factors and poorly controlled hypertension, a process similarly associated with diminished maximal flow.^31^

In functional MVD, minimal microvascular resistance during stress is preserved compared with those with preserved CFR; therefore, this measurement cannot account for the ischemic changes identified during perfusion imaging. Wave intensity analysis provides unique insight into cardiac-coronary coupling among both MVD endotypes. Whereas coronary perfusion efficiency increases or is maintained with exercise and hyperemia in the healthy heart, in MVD perfusion efficiency decreases with both forms of stress.^10^ Reduced perfusion efficiency in MVD demonstrates that greater energy needs to be expended to achieve the same degree of coronary blood flow augmentation, providing an ischemic substrate in both functional and structural MVD groups. The decreased perfusion efficiency with exercise in MVD is primarily determined by attenuated augmentation of the BEW, which is usually the main driver of coronary perfusion in health. The BEW is generated during ventricular lusitropy, thus flow acceleration will be diminished when there is diastolic dysfunction.^32^ Conversely, subendocardial hypoperfusion results in diastolic dysfunction, while transmural hypoperfusion results in overt systolic and diastolic dysfunction.^33^ While establishing causality remains challenging because of the intricate interplay of these parameters, exercise induced diastolic dysfunction and ischemia will amplify each other in a potentially deleterious cycle. In our study, both patient groups had similar resting diastolic function in terms of resting echocardiography while a trend toward increasing NT-proBNP values from patients with normal CFR, to functional MVD and structural MVD may support the notion of temporal progression of MVD disease states.

During systole, compression of the subendocardium causes retrograde filling of the subepicardial vessels, therefore antegrade subendocardial filling occurs exclusively in diastole. Myocardial oxygen demand is increased in the subendocardium, increased vasculature within this layer ensures perfusion is maintained throughout the cardiac cycle and indeed in health, pharmacological vasodilatation maintains hyperperfusion within the subendocardial layer.^34^ We have demonstrated a reduction in coronary perfusion efficiency during vasodilator hyperemia in MVD, accentuation of the BCW and attenuation of the BEW will disproportionately affect subendocardial blood flow. Loss of privileged perfusion of this layer in MVD may be the driving force behind subendocardial ischemia leading to diastolic dysfunction and inefficient coronary perfusion during exercise. Myocardial contraction in the face of higher vascular resistance within the subendocardial layer may also account for the exaggerated BCW during isovolumetric contraction, while conversely a larger BCW can diminish systolic perfusion. Indeed, the mechanics of image acquisition during perfusion CMR whereby the basal-, mid-, and apical-LV slices are acquired during mid-diastole, mid-systole, and late-diastole, respectively, demonstrates that subendocardial perfusion in MVD is impaired throughout the cardiac cycle (Table 2). The ability of the coronary microvasculature to dilate during periods of stress seems pivotal to preventing the maladaptive changes to exercise and myocardial perfusion observed within the MVD cohort. With therapies shown to improve outcome in patients with MVD as defined by low CFR, further work will need to be carried out to determine whether the distinct functional and structural MVD endotypes should be managed differently.^35,36^

### Coronary Flow Reserve and Inducible Ischemia

The Coronary Vasomotion Disorders International Study Group agreed upon the following criteria for the diagnosis of microvascular angina: (1) presence of symptoms suggestive of myocardial ischemia; (2) objective documentation of myocardial ischemia; (3) absence of obstructive CAD >50% coronary diameter reduction and/or fractional flow reserve >0.80); and (4) confirmation of a reduced coronary blood flow reserve and/or inducible microvascular spasm.^37^ We have demonstrated that a CFR<2.5 (in our NOCAD cohort) accurately predicted myocardial ischemia and exercise pathophysiology. Emerging data suggests that a binary CFR threshold of 2.5 more accurately identifies patients with MVD than the less sensitive value of 2.0, both currently accepted by international guideline committees and experts within the field.^37–40^ Whether a CFR<2.5 alone is sufficient to diagnose MVD (when noninvasive ischemia testing has not been performed) is something the guideline committees may wish to consider, but in the meantime this study should provide clinicians with a strong recommendation to perform invasive coronary physiology in patients who undergo cardiac catheterization. Recent studies have also demonstrated the correlation between global measurements of myocardial perfusion and invasive coronary functional testing.^41^ Within our study, 22% of patients with normal vasodilator reserve demonstrated inducible ischemia on quantitative perfusion imaging, and this group of patients may have other ischemic pathologies. One explanation is that CFR integrates epicardial and endocardial blood flow within a given territory; good perfusion in the former may mask ischemia affecting the latter.

### Study Limitations

This was a mechanistic single-center study with relatively small numbers of patients in each group. The lack of correction for multiplicity increases the possibility for type 1 error for each of the analyses presented. Invasive CFR is currently the most accepted method for classifying MVD in patients with NOCAD. Like all biological measurements, CFR is a continuous variable and for this study a dichotomous CFR threshold of 2.5 was adopted to define MVD, acknowledging that a lower threshold may have had enhanced specificity at the cost of sensitivity. Moreover, given that our control group consisted of patients who were not healthy volunteers but who had symptoms that had led to angiography, we adopted a 2.5 threshold to ensure that their endothelium-independent microvascular function was truly normal. Indeed, patients in the control group may have occult coronary abnormalities such as endothelial dysfunction or coronary vasospasm that could be unmasked during provocation testing. The study was powered to detect differences in exercise and myocardial perfusion physiology between patients with preserved CFR and diminished CFR, not among MVD endotypes. A larger sample size may have enabled the identification of differences between the 2 endotypes. A normal hyperemic microvascular resistance value is currently undefined in a NOCAD population because of the absence of a gold standard measure to assess microvascular function in vivo. However, our study describes 2 distinct endotypes with differing degrees of systemic disease involvement based around a 2.5 mmHg/cm/s value. We were not able to directly assess myocardial perfusion during exercise in this study; therefore, we were unable to demonstrate exercise-induced ischemia per se, although vasodilator mediated perfusion heterogeneity during CMR is widely regarded as a surrogate of ischemia in routine clinical practice. Subendocardial hypoperfusion during hyperemia represents a very early stage of the ischemic cascade and so its presence may not correlate perfectly with later stages, such as wall motion abnormalities. However, this is a widely adopted index for identifying the presence of inducible ischemia, particularly in a NOCAD population and is strongly recommended in practice guidelines. Because of the length of this protocol, not all study patients underwent CMR; however, this was factored into the study design and statistical power calculation.

## Conclusion

Patients with MVD, defined by diminished vasodilator reserve in the cardiac catheter laboratory, have inducible ischemia and inefficient coronary perfusion during exercise. These findings are consistent among the 2 distinct MVD endotypes, defined by either abnormal resting or minimal microvascular resistances and reflect differing degrees of systemic disease involvement. Preserved coronary vasodilatory reserve is pivotal to normal exercise physiology and myocardial perfusion; reduced CFR may lead to poorer clinical outcomes in patients with angina and NOCAD through these pathophysiological changes. Whether each endotype exhibits a different prognosis or requires distinct therapies merits further investigation.

## Sources of Funding

This work was supported by the British Heart Foundation (primarily through the fellowships FS/16/49/32320 and FS/13/15/30026) and by the National Institute for Health Research (through the Biomedical Research Centre award to King’s College London and Guy’s and St Thomas’ Hospital).

## Disclosures

None.
